# Metalloproteinases TACE and MMP-9 Differentially Regulate Death Factors on Adult and Neonatal Monocytes After Infection with *Escherichia coli*

**DOI:** 10.3390/ijms20061399

**Published:** 2019-03-20

**Authors:** Stephan Dreschers, Christopher Platen, Andreas Ludwig, Christian Gille, Natascha Köstlin, Thorsten W. Orlikowsky

**Affiliations:** 1 Department of Neonatology, University Children’s Hospital, Aachen 52074, Germany; sdreschers@ukaachen.de (S.D.); cplaten@ukaachen.de (C.P.); 2 Department of Pharmacology and Toxicology, University Hospital, Aachen 52074, Germany; aludwig@ukaachen.de (A.L.); natascha.koestlin@med.uni-tuebingen.de (N.K.); 3 Department of Neonatology, University Children’s Hospital, Tuebingen 72074, Germany; christian.gille@med.uni-tuebingen.de

**Keywords:** matrix-metalloproteinase, monocytes, inflammation, phagocytosis, apoptosis

## Abstract

**Background:** Cleaving ligands and receptors of the tumor necrosis factor (TNF) superfamily can critically regulate the induction of apoptosis. Matrix metalloproteinases (MMPs) such as MMP-9 and tumor necrosis factor-α-converting enzyme (TACE) have been shown to cleave CD95-Ligand (CD95L) and TNF/(TNF receptor-1) TNFR1 which induce phagocytosis induced cell death (PICD) in adult monocytes. This process is reduced in neonatal monocytes. **Methods:** Here we tested in vitro, whether *Escherichia coli* infection mounts for activation of MMP-9 and TACE in monocytes and whether this process regulates PICD. **Results:** The surface expression of TACE was most prominent on infected adult monocytes. In contrast, surface presentation of MMP-9 was highest on infected neonatal monocytes. Selective blocking of MMP-9 decreased CD95L secretion, while inhibition of TACE left CD95L secretion unaltered. Blocking of MMP-9 increased surface CD95L (memCD95L) expression on infected neonatal monocytes to levels comparable to infected adult monocytes. Moreover, MMP-9 inhibition raised PICD of infected neonatal monocytes to levels observed for infected adult monocytes. In contrast, TACE inhibition decreased PICD in infected monocytes. Addition of extracellular TNF effectively induced memCD95L presentation and PICD of adult monocytes and less of neonatal monocytes. **Conclusion:** MMP-9 activity is crucial for downregulating cell-contact dependent PICD in *E. coli* infected neonatal monocytes. By this mechanism, MMP-9 could contribute to reducing sustained inflammation in neonates.

## 1. Introduction

Perinatal mortality and morbidity is often caused by preterm delivery. Although modern intensive care has increased the number of surviving preterm infants, they may suffer from complications and lifelong handicaps. Preterm delivery can be triggered by intrauterine infections and numerous environmental and genetic factors, which give raise to an inappropriate cytokine response [1]. Granulocytes, monocytes and monocyte-derived macrophages build up the first line of defence against infections. Especially, macrophages secrete pro-inflammatory cytokines that trigger the antibacterial response. This promotes recruitment of phagocytes to the site of infection and subsequent phagocytosis and killing of the infectious pathogens. After the clearance of bacteria, the pro-inflammatory response is shifted into an anti-inflammatory situation. In this process, monocytes releasing anti-inflammatory mediators become more prominent while phagocytosing monocytes are removed via apoptosis [2]. This phagocytosis induced cell death (PICD) extenuates the pro-inflammatory response of monocytes after the acute phase of infection. Monocytes of neonates exhibit reduced PICD, resulting in a prolonged pro-inflammatory phase compared to adults [2]. There is increasing evidence that the inadequate termination of inflammation in prematurity (“sustained inflammation”) is of major impact on tissue and organ damage later in life [3].

We have previously shown that monocytes derived from neonatal cord blood (CBMO) undergo less apoptosis following infection with *E. coli* or group B-streptococci (GBS), while monocytes from adult donors (PBMO) show a strong apoptotic response to infection [4,5]. Moreover, infected PBMO were able to induce apoptosis of monocytes, which were not infected (bystander-apoptosis), thereby actively terminating inflammatory immune response [6]. The mechanisms by which cell death was induced were activation of internal and external apoptosis pathways mediated through CD95L/CD95 (receptor) [7], and apoptosis induction by tumor-necrosis-factor-alpha (TNF) via tumor-necrosis-factor-receptor-1 (TNFR1) followed by caspase-cleaving and consecutive cell death [8].

Previous work revealed that CBMO show less CD95L-expression, TNFR1-internalization and TNF secretion compared to PBMO. This difference could be the result of a distinct posttranslational regulation of these pro-apoptotic factors in adults and neonates. One possible regulatory mechanism is the limited proteolysis of surface. This process, designated as shedding, can critically reduce ligands/receptors on the cell surface and thereby down-regulate the signal transmission [9,10]. The responsible enzymes are termed ectodomain sheddases and often represent zinc containing metalloproteinases.

The most relevant metalloproteinase for shedding of TNF and TNFR-1 is the tumor necrosis factor-converting enzyme (TACE/ADAM17). TACE belongs to the ADAM family proteases that share the characteristics of type-1-transmembrane proteins containing a catalytically active metalloproteinase, a disintegrin, an EGF and a transmembrane domain. Many other substrates for TACE have been identified including the IL-6 receptor [10], CD62L [11,12] and growth factors such as AREG and EGF [13].

Cleavage of CD95L can be mediated by the matrix metalloproteinase-9 (MMP-9, Gelatinase-B). This protease is well known to cleave extracellular matrix proteins such as collagen IV and V. De novo synthesized MMP-9 (pro-MMP-9) is activated by a two-step auto-proteolysis [14,15]. Moreover, MMP-9 activity can be regulated by TNF [16] and lipopolysaccharide (LPS), therefore playing an important role in endotoxin tolerance [17].

To better understand differences in PICD between neonatal and adult monocytes we here study the regulation of MMP-9 and TACE expression in CBMO and PBMO after *E. coli* infection. By blocking experiments with distinct metalloproteinase inhibitors we then investigate the role of MMP-9 and TACE for surface-expression and secretion of death ligands in monocytes from adults and neonates. Next, we perform blocking experiments to obtain information on the involvement of metalloproteinase activities for PICD of CBMO and PBMO. Finally, we compare the relevance of cell contact formation for bystander apoptosis induced in CBMO and PBMO target cells. Our results suggest that MMP9 and TACE are differentially regulated and have opposite functions in PICD. MMP9 expression is comparatively high in CBMO and seems in part responsible for shedding of CD95L and the low rate of PICD in these cells.

## 2. Results

### 2.1. Plasma-Membrane Expression of Metalloproteinases is Induced by E. coli Infection

**Figure 1:**
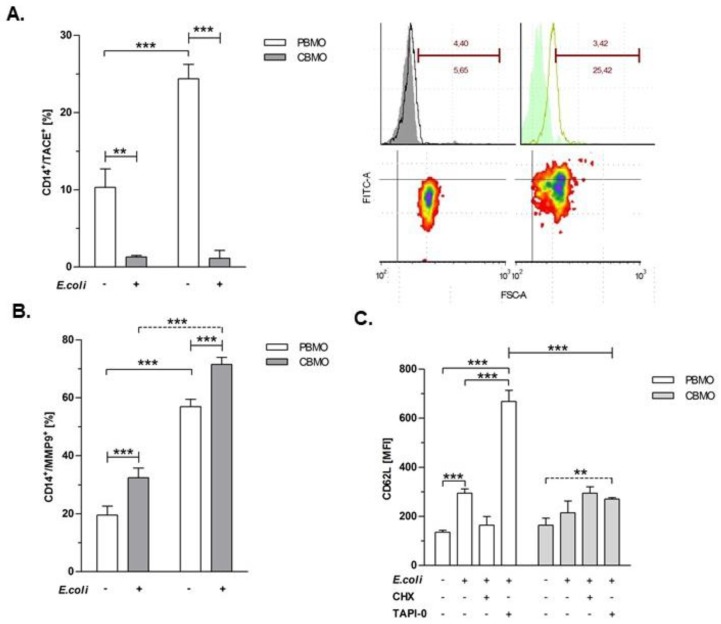
TACE and MMP-9 expression is induced four hours p.i. by infection with *E. coli*. TACE expressing monocytes were assessed before and after infection with *E. coli*. (**A**) Histogram plots to the right compare isotype controls (filled) and anti-TACE stained PBMO which were non-infected or *E. coli* infected. Density plots (below) detail the distribution of *E. coli* infected PBMO regarding TACE surface expression (compare to the non-infected PBMO to the left). *E. coli* MMP-9 expressing monocytes were assessed before and after infection with *E. coli*. (**B**) (*n* = 5, *** *p* < 0.001, forked bars represent Student’s *t*-test, blunt-ended bars represent ANOVA). Blocking of TACE activity increases its substrate CD62L. (**C**) Monocytes were infected with GFP-*E. coli* for 4 h with or without indicated inhibitors and surface expression of CD62L was determined. (**A**–**C**); (*n* = 5, ** *p* < 0.01, *** *p* < 0.001, forked bars represent Student’s *t*-test, blunt-ended bars represent ANOVA).

In previous studies we observed that phagocytosing PBMO and CBMO express similar levels cell associated TNF whereas CBMO secrete much lower levels of soluble TNF than PBMO 24 h and 48 h post-infection (p.i.), (Supplemental Figure S1). These findings led us to the question whether ectodomain sheddases responsible for release of TNF and other death ligands (e.g., CD95L) could be differentially regulated in PBMO and CBMO. We here focus on the two metalloproteinase TACE and MMP-9 known as major shedding enzymes for membrane expressed TNF (memTNF) and memCD95L, respectively. First, we studied the surface expression level of TACE and MMP9 by flow cytometry before and after infection with *E. coli.* Infection caused a significant up-regulation of TACE on PBMO, whereas the expression level remained low in CBMO (Figure 1A). In contrast to TACE, the expression level of MMP-9 was significantly higher in CBMO compared to PBMO (Figure 1B).

To check whether expression profiles were correlated to enzyme activities and to exclude the possibility that infection caused alterations in the presence of MMP-9 and TACE only coincidentally, we performed a shedding assay for CD62L as an indicator for TACE-activity (Figure 1C). CD62L plasma membrane expression was induced after *E. coli* infection in PBMO but less in CBMO. Administration of the TACE inhibitor TAPI-0 considerably increased CD62L expression on infected PBMO and less on CBMO, indicating that the expression level of TACE is reflected by the shedding of one of the major substrates. By contrast, CHX treatment, which is known to inhibit MMP-9 activity [18] had no effect on CD62L expression (Figure 1C). These data show that under our experimental conditions PBMO exhibit higher TACE expression and activity while CBMO exhibit higher expression of MMP9. Furthermore, the results show effectiveness of the used inhibitors.

### 2.2. Metalloproteinase-Inhibition Down-Regulates the Expression of CD95L and TNF

**Figure 2:**
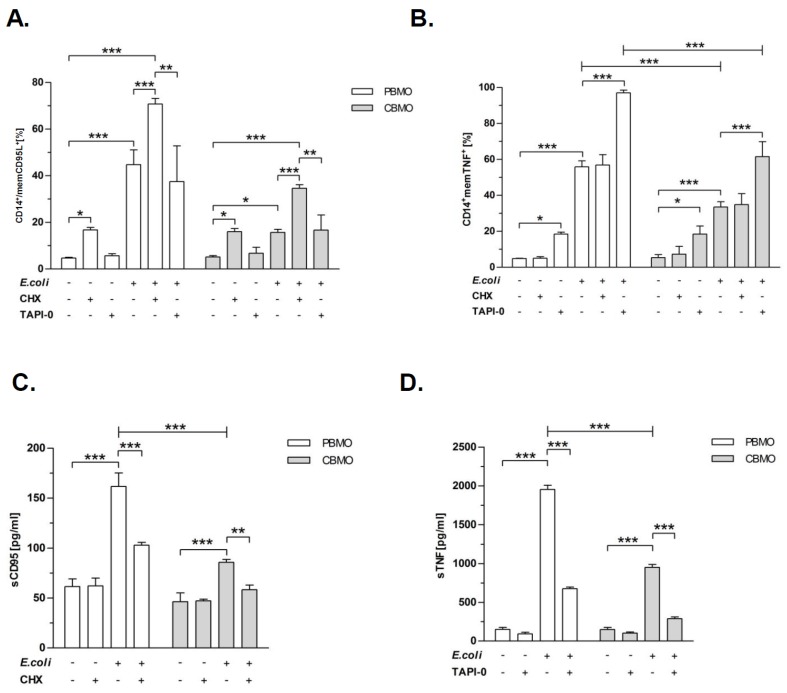
Blockage of metalloproteinases modulated expression of memCD95L and memTNF. PBMO and CBMO were infected and/or treated with the indicated metalloproteinase inhibitors. Expression of memCD95L (**A**) and memTNF (**B**) were assessed on CD14-positive gated monocytes. The same groups were analyzed for secreted (s) CD95 (**C**) and TNF (**D**) 24 h p.i. via ELISA (*n* = 5, * *p* < 0.05, ** *p* < 0.01, *** *p* < 0.001, forked bars represent Student’s *t*-test, blunt-ended bars represent ANOVA).

Next we checked whether TACE and MMP-9 activities on *E. coli* infected monocytes could affect the expression of the two reported substrates CD95L and TNF, respectively. To this end, we added the metalloproteinase inhibitors TAPI-0 and CHX to infected monocytes (Figure 2). Addition of these inhibitors had no effect on the phagocytosis index under the chosen conditions (Supplemental Figure S2).

PBMO showed reduced levels of memCD95L after treatment with both inhibitors and an increase of memCD95L after *E. coli* infection. CBMO showed no difference regarding memCD95L levels. The memTNF levels increased significantly after *E. coli* infection in PBMO and CBMO (Supplemental Figure S3). Inhibition of MMP-9 by CHX was associated with a two-fold increase of memCD95L on PBMO and CBMO, whereas TACE inhibitor TAPI-0 had no effect (Figure 2A, compare columns 1, 2 and 3). The results are in line with the CD62L shedding assay, which shows a clear specificity of CHX for MMP-9 (Figure 1B). Infection with *E. coli* increased the number of memCD95L-positive PBMO eight-fold. This effect was much lower (four-fold) in CBMO (Figure 2A, compare columns 1 and 4). CHX but not TAPI-0 increased the number of memCD95L expressing infected PBMO. CHX also increased the number of memCD95L expressing CBMO to levels that were observed for infected but not CHX treated PBMO (Figure 2A, compare columns 4 and 11).

CHX had no effect on the expression of memTNF. However, treatment with TAPI-0 clearly increased the number of memTNF expressing PBMO and CBMO (Figure 2B, compare columns 1 and 3). Infection with *E. coli* amplified the expression level of memTNF three-fold on PBMO and two-fold on CBMO (Figure 2B, *p* < 0.05 vs. non-treated, non-infected monocytes). After blockage of TACE, nearly all PBMO expressed memTNF, while only 60% of CBMO did (Figure 2B, compare columns 4 and 6).

The results mirrored the effects of CHX and TAPI-0 on memCD95L and memTNF: While increasing memCD95L on infected monocytes (Figure 2A), CHX significantly diminished the concentration of secreted CD95L (Figure 2C). Similar results were obtained for TAPI-0 with respect to secreted TNF (Figure 2D).

### 2.3. Inhibition of TACE Reduced PICD, but Inhibition of MMP-9 Restored PICD in CBMO

**Figure 3:**
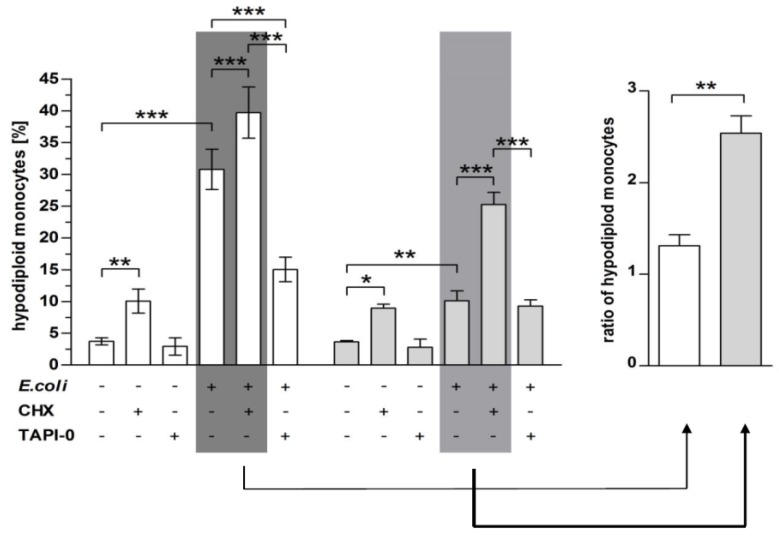
Blockage of MMP-9 increased PICD of CBMO. PBMO and CBMO were infected and/or treated with the indicated metalloproteinase inhibitors. Apoptosis was determined by Nicoletti assay 24 h p.i. The right panel gives the ratio of the values highlighted in dark-grey and grey, respectively (*n* = 5, * *p* < 0.05, ** *p* < 0.01, *** *p* < 0.001, forked bars represent Student’s *t*-test, blunt-ended bars represent ANOVA).

Since our experiments indicated the involvement of metalloproteinases in the regulation of death receptors and ligands we addressed the question as to whether inhibition of metalloproteinases affects the induction of apoptosis in uninfected and infected monocytes (Figure 3). Treatment with CHX doubled apoptosis rates in uninfected PBMO and CBMO (compare columns 1 and 2). TAPI-0 did not show this effect (compare columns 1 and 3). In concordance with our previous results, PICD of CBMO was diminished compared to PBMO (column 4). Treatment with CHX increased the PICD in infected PBMO significantly by about 10%. In infected CBMO, the effect of CHX on PICD was much higher and doubled PICD rates on *E. coli*. infected monocytes. PICD rates of CHX treated infected CBMO were two times higher than infected PBMO (Figure 3, right panel) In contrast, TAPI-0 addition reduced PICD in PBMO by about 50% (*p* < 0.05 vs. infected PBMO). In CBMO PICD was found to be unaltered (colums 6).

### 2.4. TNF and E. coli Infection Triggers Expression of memCD95L

**Figure 4:**
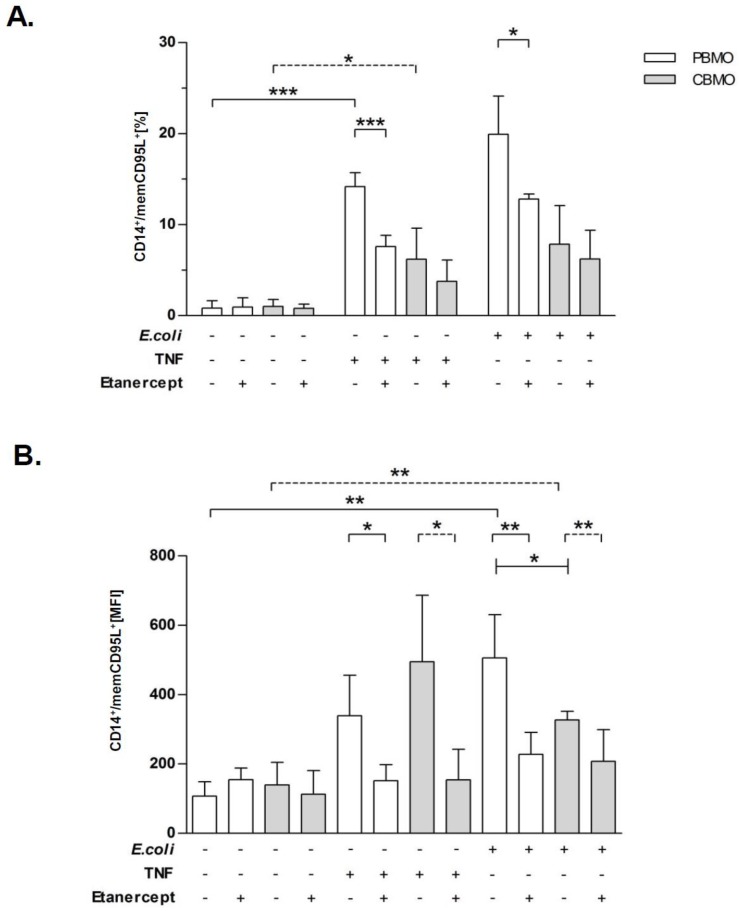
TNF triggers expression of CD95L. PBMO and CBMO were stimulated with TNF and/or infected with *E. coli* for four hours and analyzed for CD95L expression. Some groups were pre-treated with etanercept. The number of PBMO and CBMO expressing memCD95L were determined as the percentage (**A**) and the mean concentration given as the mean intensity (MFI), (**B**); for A and B, *n* = 5, * *p* < 0.05, ** *p* < 0.01, *** *p* < 0.001, forked bars represent Student’s *t*-test, blunt-ended bars represent ANOVA.

We had observed that infected PBMO and CBMO release considerable amounts of soluble TNF in a manner that most likely involves TACE (Figure 2D). Moreover, this inhibition was associated with a reduction of CD95L expression (Figure 2A). This may suggest that a cross-talk between TNF production and memCD95L expression exists. To address this possibility, monocytes were either incubated with soluble recombinant TNF and/or infected with *E. coli* and memCD95L expression was analyzed. Both addition of TNF and *E. coli* infection strongly up-regulated memCD95L on PBMO and to a lesser degree in CBMO (Figure 4, compare groups 2 and 3). As an inhibitor of TNF, we applied the TNF inhibitor etanercept to one group (Figure 4A, columns 2 and 4) resulting in considerable reduction of CD95L expression in PBMO, while the effect was not significant in CBMO. Furthermore, pre-treatment with etanercept reduced apoptosis rates significantly in TNF-incubated and *E. coli-*infected PBMO, but had no effect on CBMO (Figure 4A). Comparing the mean intensity values (MFI) of memCD95L (Figure 4B) reflected the specific effect of TNF, since addition of etanercept reduced the concentration of memCD95L on both PBMO and CBMO after TNF treatment and infection, respectively.

### 2.5. Apoptosis of Bystander Monocytes is Reduced Without Cellular Contact

**Figure 5:**
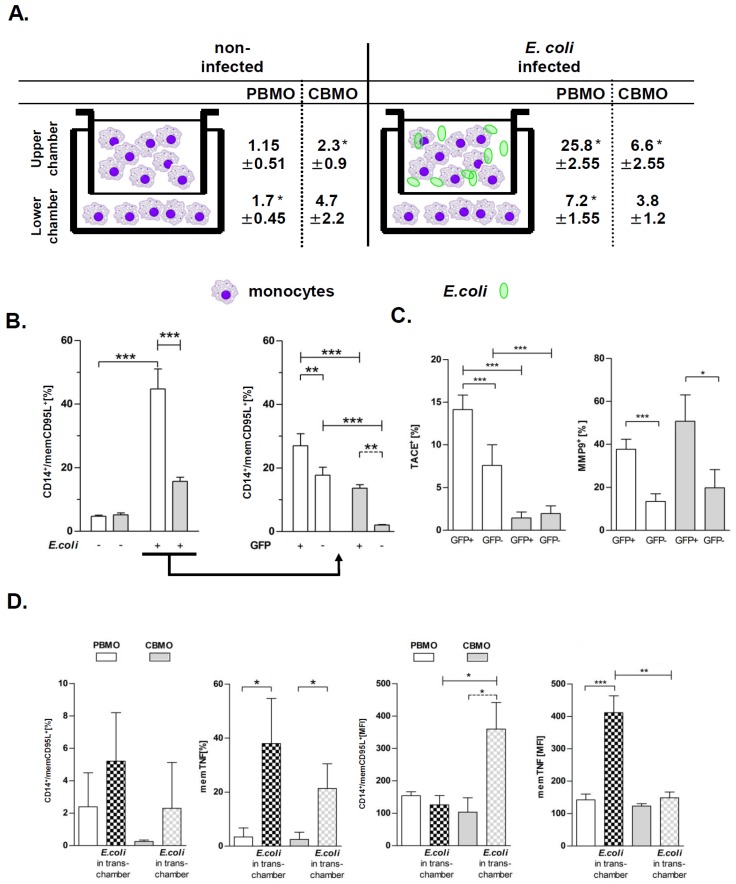
Monocyte PICD is enhanced by cell–cell contact. Monocytes were infected with GFP-*E. coli* for one hour, washed and re-incubated for another 24 h in transwell chambers (experimental setup sketch); non-infected cells served as controls. Monocytes from the same donor were co-cultivated in compartments, separated by teflon membranes. Apoptosis was detected by hypodiploid DNA-content (**A**, *n* = 6 for PBMO and *n* = 4 for CBMO; * *p* < 0.05). PBMO and CBMO were sub-gated (**B**) in phagocyting/binding (GFP^+^) and non-phagocyting (GFP^−^) monocytes and analyzed for memCD95L expression. Subgated PBMO and CBMO were further analyzed for TACE (**C**, left panel) and MMP9 (**C**, right panel) expression. (**D**) The expression of memCD95L and memTNF of monocytes in the lower (“trans“) chamber was assessed. The percentage of surface expressing monocytes (panels to the left) and the mean values (MFI, panels to the right) were determined (*n* = 5, * *p* < 0.05, ** *p* < 0.01, *** *p* < 0.001, forked bars represent Student’s *t*-test, blunt-ended bars represent ANOVA).

The surface expression levels of TNF and CD95L are separately regulated by TACE and MMP-9 leading to the release of soluble mediators. This process may affect bystander apoptosis in which cells could be directed to undergo apoptosis by either cell-to-cell contact or soluble mediators. In fact, bystander-apoptosis in PBMO required intercellular contact with phagocytosing monocytes, as we have previously shown in a transwell chamber setup [8]. To compare this aspect in CBMO and PBMO, we infected monocytes with *E. coli*-GFP in one (upper) transwell chamber and co-cultivated non-infected monocytes of the same donor in the other (lower) chamber (Figure 5A, sketch). In the upper chamber apoptosis occurred in PBMO in a significantly higher percentage compared to CBMO, as was expected. In the lower chamber, apoptosis of PBMO was reduced by 70% compared to PBMO in the upper chamber (Figure 5A). In CBMO, contact-independent (“trans“) apoptosis was nearly absent.

We then questioned whether the expression of death receptors or shedding enzymes on cells that had either bound or ingested *E. coli* would be different from that on those cells, that had no contact to bacteria. As previously reported [6], we distinguished monocytes that had either bound or ingested *E. coli* (GFP-positive, GFP^+^) from those that had no contact with bacteria (GFP-negative, GFP^−^). CD95L was significantly higher up-regulated on the surface of GFP^+^ PBMO as compared to GFP^−^ PBMO (Figure 5B). For CBMO, the upregulation of CD95L on GFP^+^ cells was less compared to on GFP^+^ PBMO (*p* < 0.005). Moreover, for on GFP- cells much less upregulation of CD95L was seen on CBMO than on PBMO.

Since CD95L expression can be regulated via TACE through TNF signalling and via MMP9 by cleavage of CD95L we analyzed TACE and MMP-9 expression on GFP^+^ and GFP^−^ PBMO and CBMO, respectively. GFP^+^ PBMO expressed more TACE and MMP-9 compared to GFP^−^ PBMO (Figure 5C, left panel, *p* < 0.001). MMP-9 was also upregulated on GFP^+^ CBMO compared to GFP^−^ CBMO (Figure 5C, right panel). By contrast TACE expression was very low and comparable on GFP^+^ and GFP^−^ CBMO (Figure 5C). These results suggest that CBMO are less effective in inducing bystander apoptosis than PBMO. This correlates with less upregulation of CD95L on CBMO compared to PBMO target cells, which is again associated with a low expression of TACE but still high expression of MMP-9 on CBMO compared to PBMO target cells. This correlation further supports our notion that activity of MMP-9 rather than of TACE contributes to reduced levels of PICD that are typically observed in neonate monocytes.

Additionally, the percentage of memCD95L-, memTNF-positive PBMO as well as the density of memTNF on PBMO was higher than on CBMO. In contrast, with less memCD95L-positive CBMO, their mean density was higher than on PBMO (Figure 5D).

## 3. Discussion

The present study compared the induction of PICD in infected and non-infected monocytes (bystander cell death) from cord blood and adult blood, utilizing an in vitro *E. coli* infection model with respect to a distinct regulation pattern of the metalloproteinases TACE and MMP-9 (Figure 1). MMP expression was distinctly regulated by infection in PBMO and CBMO. Whereas infected CBMO exhibited less TACE expression and activity, they reacted with stronger expression of MMP-9 (Figure 1) as compared to PBMO. Depending on the expression pattern of MMP-9 and TACE, death ligands CD95L and TNF were either presented on the surface or shedded by distinct monocyte populations and thereby functionally controlled in regard to PICD (Figure 2, Supplemental Figure S3). We speculate that this regulation may be phagocytosis dependent (see Figure 5B), since the percentage of memCD95L appears higher on GFP+ phagocytosing monocytes (Figure 5B) than on non-phagocytosing (GFP-) monocytes. This observation was one of the reasons for monitoring the shedding of memCD95L and TNF via ELISA (Figure 2C,D). We could show, that PICD in CBMO was diminished (Figure 3), confirming earlier results [4]. TNF secretion triggered expression of memCD95L on PBMO but critically less on CBMO (Figure 4) which may explain that induction of PICD is predominantly contact-dependent (Figure 5). The data suggest that phagocytosis regulates expression of MMP-9 but not TACE in *E. coli* infected CBMO (Figure 5). To our knowledge, data on the expression and activity of metallomatrixproteinases in CBMO have been scarcely published.

The results of the actual study indicate an interlocked action of TNF and memCD95L. They suggest a functional model, where infection with *E. coli* activates TNF and consecutive TACE secretion in PBMO. TACE cleaves memTNF allowing predominantly ligation to TNFR1, since soluble TNF preferentially binds to TNFR1 [19,20]. The TNFR1/TNF ligand receptor complex is internalized to induce apoptosis [8,20]. This is in line with the findings that TAPI-0 decreases concentrations of soluble TNF resulting in reduced apoptosis (Figure 2B,D and Figure 3) However, our experiments provide evidence that soluble TNF also induces memCD95L (Figure 4). Pro-apoptotic signaling of CD95L/CD95R (receptor) can be distinguished from death receptor signaling by acting in a cell–cell contact dependent manner [21]. Increasing the amount of memCD95L by the MMP-9 inhibitor CHX is accompanied by an increase in apoptosis rates (Figure 2A,C and Figure 3).

This model is further supported by the low abundance of secreted TNF, TNFR1 [6] and CD95L in CBMO resulting in reduced PICD [4]. It explains also the significantly reduced trans-activation of PICD in PBMO and CBMO (Figure 5A). Phagocytosing monocytes express more TNF (Supplemental Figure S1A) which may cause a higher level of memCD95L in GFP^+^ monocytes (Figure 5B). Previous data have shown that blockage of TNF almost abolished PICD in PBMO [6]. The fact that CBMO secrete significantly less TNF than PBMO (Supplemental Figure S1B), but TNF stimulation increases the memCD95L-expression (Figure 4) may explain the observation of a reduced PICD initiation in cord blood (Figure 3). However, PBMO and CBMO showed a different reaction to factors which were secreted by phagocytosing mates in the transwell experiments (Figure 5D), pointing to a fundamental different meaning of cell-contact independent signaling in neonatal monocytes. Future experiments will elucidate these differences.

Various microbial, fungal and viral agents such as *Streptococcus pneumoniae* [22], zymosan [23] and HIV [24] were reported to initialize bystander apoptosis in monocytes. For resolving inflammation, bystander apoptosis may in the same way be essential [25]. Disbalancing this system may result in overwhelming infection or prolonged inflammation, thus the reduced bystander apoptosis of CBMO may be critical for the resulting immune reaction.

The mechanism of infection induced MMP-9/TACE expression should be clarified by future experiments. It was recently published, that soluble CD95L as well as memTNF activate the NFκ-B pathway which in turn targets metalloproteinases such as MMP-9 [26]. TACE, which also was examined in this study, is activated by infection-induced p38 MAP kinase and reactive oxygen species (ROS) [27].

MMP-9 and TACE are integrative members of a feedback control system which can skew the signal transduction of TNF-α and CD95L from pro-apoptotic to pro-inflammatory by balancing soluble and plasma-membrane bound forms and vice versa [28,29]. Reduced TACE activity was shown to promote endotoxin tolerance [30] without engagement of TLR4 [31], which circumvents an overwhelming inflammation upon infection.

The damage of organ tissues can be curtailed by controlling the expression of MMP-9. Enhanced expression of MMP-9 was correlated to COPD [32,33]. MMP-9 activation plays also a role in amniotic membrane rupture during labor [34] and enhanced MMP levels were associated with sepsis [35].

In this study we attributed a co-expression of MMP-9 and TACE with memCD95L and memTNF to the induction of apoptosis and proved this by simultaneous exposure to inhibitors. As a limitation it has to be mentioned that substrate specificity of the two enzymes is still controversial. The cleavage of memTNF by TACE is well documented [36,37] but the cleavage of the receptor TNFR1 in vivo has been discussed [38]. Furthermore it was reported that pathogenic *E. coli* synthesize proteins with metalloproteinase like properties [39,40] which could contribute to the shedding of TNF and CD95L. The results of our study are based on relatively small sample sizes and did not follow up on kinetics of cord and peripheral blood of newborns longitudinally. The neonatal situation several days postnatal is therefore not properly reflected by this study.

PICD and bystander apoptosis represent important tools to shape the immune response upon infection. Our data suggest phagocytosis-related cell death of non-phagocytosing monocytes, designated as monocytic bystander kill after infection with *E. coli*. It provides evidence that if monocytes are “at the wrong place at the wrong time”, i.e., near or in contact to phagocytosing monocytes, they may be killed by fratricide. To what extent this may influence the course of neonatal sepsis, remains to be elucidated in vivo.

## 4. Materials and Methods

### 4.1. Patients

The study protocol was approved by the Ethics Committees of Aachen University Hospital (Permission No: EK150/09, 6 October 2009, signed by Profs G. Schmalzing and U. Buell, respectively). All adult participants involved gave written consent to use their blood samples. All term neonates were delivered spontaneously and did not exhibit signs of infection, as defined by clinical status, white blood cell count and C-reactive protein. Mothers with amnion infection and prolonged labour (>12 h) were excluded. Umbilical cord blood was placed in heparin-coated tubes (4 IE/mL blood), immediately following cord ligation as described before [7].

### 4.2. Bacteria

#### *E. coli*-GFP

*E. coli* DH5α, an encapsulated K12 laboratory strain, carrying the green fluorescent protein (*gfp*)-mut2 gene (*E. coli*-GFP) was a generous gift from Prof. Dr. Dehio (University of Basel, Switzerland) and was used for phagocytosis as previously described. Bacteria were freshly grown in Lennox-L-Broth-medium (Invitrogen) until early logarithmic growth, resuspended in phosphate-buffered-saline (PBS) and used immediately. Infection was performed at a multiplicity of infection (MOI) of 25 which was achieved by dilution with PBS. The phagocytosis assays were performed as described [7]. The phagocytosis index (CD14^+^GFP^+^ monocytes: CD14^+^ monocytes) was analyzed by flow cytometry. In some indicated experiments, *E. coli*-GFP was replaced by *E. coli*-EOS-FP (Supplemental Figure S1).

### 4.3. Reagents

Antibodies to CD14 (clone MEM18), MMP-9 (clone 56129), TACE (clone FAB9301P), CD62L (L-selectin; clone LT-TD180) and Ig-matched controls (IgG1, IgG2b) were purchased from R&D Systems and Immunotools (Abingdon, UK and Friesoythe, Germany), respectively. FITC-labeled CD95L antibody (clone SB93a) was purchased from SouthernBiotech (Birmingham, USA) and corresponding mouse IgG2b kappa isotype control (clone eBMG2b) was obtained from eBioscience (Waltham, USA). The secondary anti-mouse-PB antibody (F’ab fragment) was purchased from Invitrogen (Darmstadt, Germany). Propidium iodide (PI), isopropyl-β-D-thiogalactopyranoside (IPTG) and antibiotics were purchased from Sigma (Munich, Germany). For the blocking of TNF an anti-TNF antibody (a chimeric molecule combining the ligand-binding domain of the TNF-receptor 2 and the Fc-domain of human IgG1 (Etanercept, Pfizer–Wyeth, Hamburg, Germany), final concentration 1 µg/mL) was administered 1 h prior to infection. TACE inhibitor N-(R)-(2-(Hydroxyaminocarbonyl)Methyl)-4-Methylpentanoyl-L-Naphthylalanyl-L-Alanine amide (TAPI-0; Merck Millipore, Darmstadt, Germany) was added to a final concentration of 100 nm/L 30 min prior to infection. PBMO and CBMO were pre-incubated with MMP-9 inhibitor CHX (chlorhexidine; Santa Cruz Biotechnologies, Heidelberg, Germany) to a final concentration of 20 µg/mL 1 h prior to the infection. This concentration was chosen since it had been previously shown to be effective in MMP-9 inhibition [18].

TNF was purchased from eBiosciences (eBiosciences-Natutec, Frankfurt, Germany), aliquoted freshly after dilution in PBS and used in apoptosis induction assays in final concentrations of 5 ng/mL.

### 4.4. Mononuclear Cell Cultures

Peripheral blood cells from adults and cord blood mononuclear cells (PBMC and CBMC) were isolated by density gradient centrifugation on Ficoll cushions (Amersham, Freiburg, Germany) as described previously [7]. Washed cells were resuspended in VLE RPMI-1640 (Biochrom, Berlin, Germany). For analysis of post-phagocytic reactions, cells were counted in an ultraplane Neubauer hemocytometer, placed at 2 × 10^6^ cells/ml in flat bottom 24 well cell culture plates (Costar, Bodenheim, Germany), containing 10% heat-inactivated fetal calf serum (FCS, Biochrom) and were incubated at 37 °C.

### 4.5. Flow Cytometry

A daily calibrated FACS-Canto flow cytometer (Becton Dickinson, Mountain View, CA, USA) was used to perform phenotypic analysis. To prevent nonspecific binding, cells were incubated with 10% fetal calf serum on ice for 10 min before staining with appropriate fluorophore coupled secondary antibodies, or isotype-specific immunoglobulin-labelled monoclonal antibodies for 20 min over ice in the dark. Monocytes were gated by forward (FSC), side scatter (SSC), and CD14 expression. For intracellular cytokine staining, monocytes were fixed in 2% paraformlaldehyde/PBS for 30 min at room temperature (RT) and washed three times with PBS. Afterwards monocytes were permeabilized utilizing a permeabilization buffer (purchased from Thermo Fisher Scientific, Hennef, Germany) according to the manufacturers’ recommendations. Data was analyzed using the FCS Express V4.0 research Edition software (DeNovo Software, Glendale, California, USA).

### 4.6. Detection of Hypodiploid Nuclei

DNA fragmentation was assessed according to Nicoletti and previously described [4]. In brief, washed cells were slowly resuspended in 2 mL of −20 °C ethanol 70% with continuous vortexing and stored for four hours at −20 °C. Cells were washed twice, resuspended in 50 µL PBS containing 13 units RNAse (DNAse free; Sigma, Taufkirchen, Germany) and incubated for 15 min at 37 °C. 180 µL of PI (70 µg/mL) was added, incubated for 20 min and analysis was performed immediately. Alternatively, mononuclear cells were stained with CD14 antibody for 15 min at RT to identify monocytes. A fixation with paraformaldehyde (2% *v/v* in PBS) for 2 h at RT replaced the ethanol fixation. Afterwards, cells were permeabilized by incubation in PBS-T (PBS, Triton X-100 0.1% *v/v*) for 20 min at RT, washed twice in PBS, resuspended in PBS-PI (PBS, 70 µg/mL PI and 13 units RNAse) and incubated for 10 min at RT before analysis by flow cytometry. Cell-doublets were discriminated by assessment of PI-width/PI-area.

### 4.7. Transwell Experiments

Transwell plates (pore diameter 0.4 µm, purchased from Corning, NY, USA) were used. Cells in the upper chamber were untreated or infected as described above. Cells separated by the Teflon membrane had no contact to bacteria as assessed by plating on appropriate growth-medium and FACS analysis.

### 4.8. ELISA

The TNF enzyme-linked immunosorbent assay (ELISA) was purchased from eBiosciences (eBiosciences-Natutec, Frankfurt, Germany) and the CD95L ELISA from Hölzel Diagnostika (Hölzel Diagnostika Handels GmbH, Cologne, Germany). Both were used according to the manufacturer’s recommendations. The read-out was executed in a spectra max 340PC ELISA reader (Molecular Devices, Sunnyvale, CA, USA) with a sensitivity from 4–500 pg/mL.

### 4.9. Statistical Analysis

Results are expressed as mean +/− standard deviation. Error bars represent standard deviations. Values of *p* < 0.05 were considered as significant. Analyses were done with statistical software (performing two-way ANOVA adjusted according to Bonferroni-Holm for multiple group comparisons as provided by GraphPad Software Statistical Package, La Jolla, CA 92037 USA).

## 5. Conclusions

Matrix metalloproteases regulate the expression of death ligands and their receptors. On neonatal monocytes CD95L density is reduced upon infection due to lower activity of MMP-9. Enhancing MMP-9 expression could be a target to restore PICD in neonatal monocytes. 

## Supplementary Materials

Supplementary materials can be found at https://www.mdpi.com/1422-0067/20/6/1399/s1.
