# Supplementary material for: Metalloproteinases TACE and MMP-9 Differentially Regulate Death Factors on Adult and Neonatal Monocytes After Infection with Escherichia coli
Source: Int J Mol Sci. 2019 Mar 20;20(6):1399. doi: 10.3390/ijms20061399 (PMC6471605; doi:10.3390/ijms20061399)
Supplement: Supplementary file 1 [file ijms-20-01399-s001.pdf]

Supplemental Figure S1

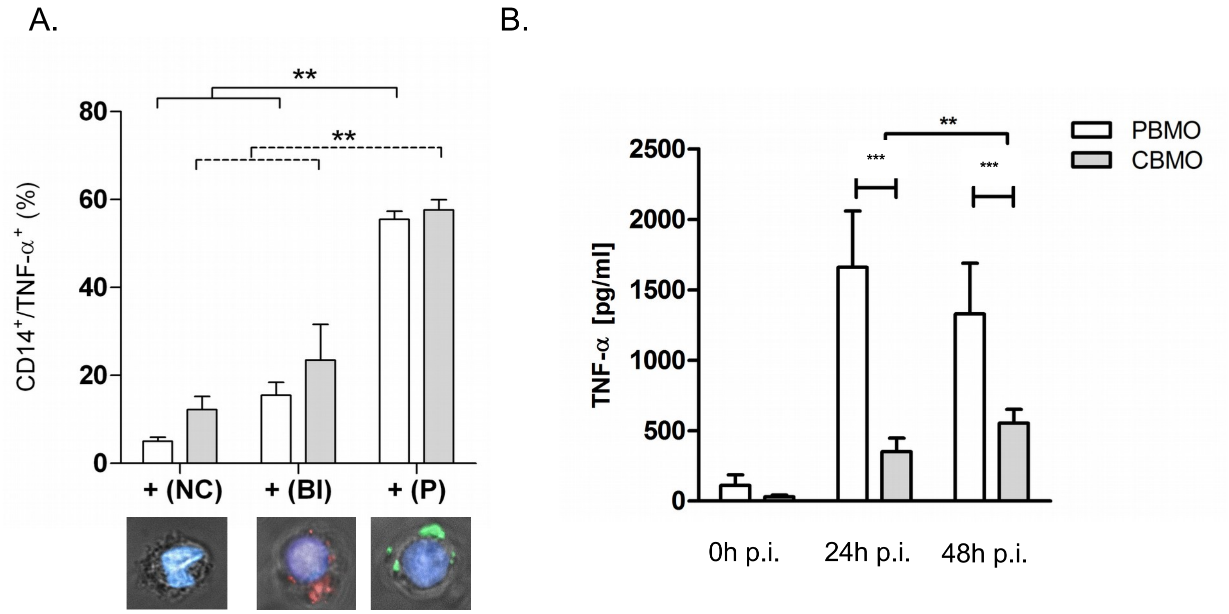

Supplemental Figure S1: Intracellular TNF production but not secretion after *E.coli* infection is comparable in PBMO and CBMO.

Monocytes were infected with EOS-FP-*E.coli* for 4 hours. Attachment and phagocytosis of *E.coli* could be discriminated by emission band shift (see micrographs below the chart). Supplementary Figure 1A represents TNF production detected by intracellular cytokine staining of infected monocytes without contact (NC), binding to *E.coli* or ingesting *E coli* (BI) and phagocyting *E.coli* (P). TNF secretion detected by ELISA in monocytes left non-infected and *E.coli* infected for 24h and 48h as inducated (n = 3; ** $p < 0.01$, *** $p < 0.001$, forked bars student`s t-test, blunt-ended bars ANOVA).

Supplemental Figure S2

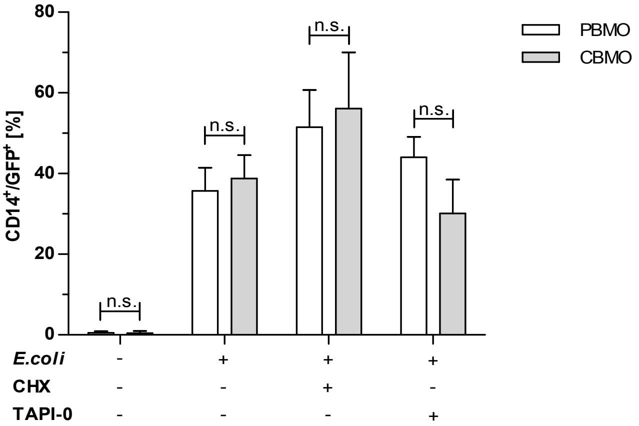

Supplemental Figure S2: Metalloproteinaase inhibition does not interfere with phagocytosis.

Monocytes were infected with GFP-*E.coli* for 4 hours and free bacteria were removed. The phagocytosis index was determined as described in material and methods.

Supplemental Figure S3

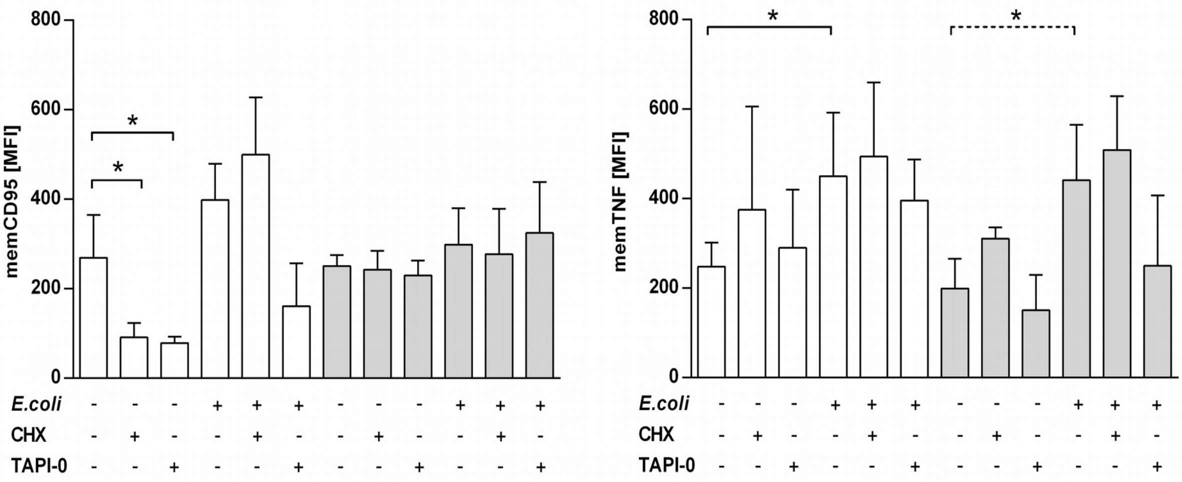

Supplemental Figure S3: Metalloproteinase inhibition interferes with memCD95 and memTNF expression.

PBMO and CBMO were treated as indicated. The mean values (MFI) of memCD95 and memTNF were assessed by FACS analysis (n = 5; * $p < 0.05$, student`s t-test).
